# Ganglionated plexi stimulation induces pulmonary vein triggers and promotes atrial arrhythmogenecity: *In silico* modeling study

**DOI:** 10.1371/journal.pone.0172931

**Published:** 2017-02-28

**Authors:** Minki Hwang, Byounghyun Lim, Jun-Seop Song, Hee Tae Yu, Ah-Jin Ryu, Young-Seon Lee, Boyoung Joung, Eun Bo Shim, Hui-Nam Pak

**Affiliations:** 1 Division of Cardiology, Yonsei University Health System, Seoul, Republic of Korea; 2 Department of Mechanical and Biomedical Engineering, Kangwon National University, Chuncheon, Kangwon-do, Republic of Korea; Universiteit Gent, BELGIUM

## Abstract

**Background:**

The role of the autonomic nervous system (ANS) on atrial fibrillation (AF) is difficult to demonstrate in the intact human left atrium (LA) due to technical limitations of the current electrophysiological mapping technique. We examined the effects of the ANS on the initiation and maintenance of AF by employing a realistic *in silico* human left atrium (LA) model integrated with a model of ganglionated plexi (GPs).

**Methods:**

We incorporated the morphology of the GP and parasympathetic nerves in a three-dimensional (3D) realistic LA model. For the model of ionic currents, we used a human atrial model. GPs were stimulated by increasing the I_K[ACh]_, and sympathetic nerve stimulation was conducted through a homogeneous increase in the I_Ca-L_. ANS-induced wave-dynamics changes were evaluated in a model that integrated a patient’s LA geometry, and we repeated simulation studies using LA geometries from 10 different patients.

**Results:**

The two-dimensional model of pulmonary vein (PV) cells exhibited late phase 3 early afterdepolarization-like activity under 0.05μM acetylcholine (ACh) stimulation. In the 3D simulation model, PV tachycardia was induced, which degenerated to AF via GP (0.05μM ACh) and sympathetic (7.0×I_Ca-L_) stimulations. Under sustained AF, local reentries were observed at the LA-PV junction. We also observed that GP stimulation reduced the complex fractionated atrial electrogram (CFAE)-cycle length (CL, p<0.01) and the life span of phase singularities (p<0.01). GP stimulation also increased the overlap area of the GP and CFAE areas (CFAE-CL≤120ms, p<0.01). When 3 patterns of virtual ablations were applied to the 3D AF models, circumferential PV isolation including the GP was the most effective in terminating AF.

**Conclusion:**

Cardiac ANS stimulations demonstrated triggered activity, automaticity, and local reentries at the LA-PV junction, as well as co-localized GP and CFAE areas in the 3D *in silico* GP model of the LA.

## Introduction

Atrial fibrillation (AF) is a cardiac rhythm disorder that leads to the absence of normal atrial contraction. Although the mechanisms of AF remain unclear, they are often divided into 2 categories: “initiation” and “maintenance” mechanisms. One of the most common trigger mechanisms of AF involves the ectopic beats that originate from the pulmonary vein (PV). With regard to maintenance mechanisms for AF, the involvement of rotating spiral waves termed as “rotors” was proposed [1]; however, contradictory evidence has been reported regarding the presence and role of these rotors in AF [2, 3]. Cardiac autonomic nerves and ganglionated plexi (GPs) are known to play an important role in the initiation and maintenance mechanisms of AF [4]. With regard to trigger mechanisms for AF, late phase 3 early after-depolarization (EAD) has been found to initiate AF [5], which was observed when the elevated intracellular calcium concentration was coupled with a shortened action potential duration (APD) [5]. As the APD of a PV cell is shorter than that of the left atrium (LA), the PV has favorable conditions for the occurrence of late phase 3 EAD following APD shortening by acetylcholine (ACh) stimulation. However, most of these electrophysiological mechanisms have been identified in animal experiments, and the role of the autonomic nervous system (ANS) in AF remains difficult to demonstrate in the intact human atrium. In the present study, we aimed to examine the role of the ANS in AF initiation and maintenance by using a realistic computer simulation of human atrial cells and LA geometry, incorporated with GPs, based on the octopus model [6]. Cardiac parasympathetic and sympathetic stimulations were simulated by increasing the I_K[ACh]_ at the GP area and by homogeneous increments in the intracellular calcium concentration, respectively [7, 8]. We also attempted to study the effect of the ANS on wave dynamics, co-localization of GP and complex fractionated atrial electrogram (CFAE) areas, and virtual ablation of GPs.

## Methods

### Two-dimensional simulation

The human atrial action potential model developed by Courtemanche et al. [9] was used as the model of ionic currents. To model the electrophysiological properties of the PV cells, the ionic currents were adjusted as described by Cha et al. [10]. The model of the ACh-activated potassium current (I_KACh_) developed by Kneller et al. [7] was incorporated in the model of ionic currents. The ACh concentration was set as 0.05 μM in order to model parasympathetic stimulation. Among the ionic currents, the sodium-calcium exchanger (NCX) and Na^+^ current (I_Na_) are known to play important roles in the occurrence of EAD [5, 11, 12]. Another component that affects the occurrence of EAD is sympathetic stimulation [8]. To examine the ionic current conditions under which EAD can be observed in the present model, we tested a range of adjusted values for NCX, I_Na_, and L-type calcium current (I_CaL_; for the incorporation of sympathetic stimulation). The L-type calcium current (I_CaL_) was increased 7-fold to simulate the effect of sympathetic stimulation [8]. Experimental studies showed that the perfusion of isoproterenol (1μM) increased the I_CaL_ by 5–8 fold [13, 14]. The NCX current was increased by 2-fold to enhance the Na^+^ influx in the presence of increased intracellular calcium. The inactivation curve of I_Na_ was shifted by +10mV to increase the voltage range of Na^+^ influx. This shift delays the closing of the inactivation gate of the Na^+^ channel, and thus increases the probability of Na^+^ influx. The computational domain consisted of 20 × 20 elements, with the size of each element being 250 μm × 250 μm. The reaction-diffusion equation for cardiac wave propagation was solved numerically [15] to determine the time-dependent changes in membrane potential at each cell in the model. Pacing was applied at a side of the computational domain 200 times with a pacing cycle length (PCL) of 100 ms; subsequently, the PCL was changed to 1000 ms. The PCL change from 100 ms to 1000 ms was applied to simulate a normal rate after the spontaneous termination of AF. In the clinical setting, paroxysmal AF is occasionally reinitiated after the spontaneous termination of AF [5]. One of the purposes of the present study was to investigate the mechanism of AF re-initiation after spontaneous termination of paroxysmal AF. Each pacing elicited depolarization after 190 ms (1:1 capture). Hence, the action potential curve, ionic currents, and intracellular ionic concentrations were examined for any irregular depolarization.

### Three-dimensional atrial model

**Fig 1:**
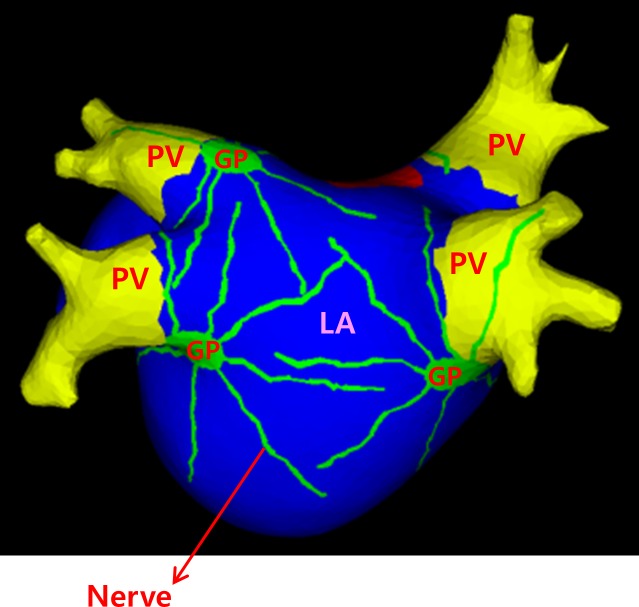
Model of GP and nerves based on the “octopus hypothesis” [6]. The PVs and LA area are indicated in yellow and blue, respectively. The GP and nerves are indicated in green.

A realistic model of the LA was constructed from human computed tomography images by using the NavX system (St. Jude Medical Inc., Minnetonka, MN, USA). A triangular mesh comprising approximately 450,000 triangles was generated in the atrial model. The human atrial action potential model used in the two-dimensional (2D) model was used for the model of ionic currents. The reaction-diffusion equation for cardiac wave propagation was solved numerically to obtain time-dependent action potential maps [16]. The effect of ACh was incorporated by modeling the GPs and the nerves based on the “octopus hypothesis” [6]. In the octopus hypothesis, the nerves are radially distributed from the GP (Fig 1). Consequently, the concentration of ACh is higher adjacent to the GP area [6]. The locations of 4 GPs (left inferior, right anterior, right inferior, and left superior GPs) were determined based on the electrophysiological mapping data of Katritsis et al. [17]. ACh was modeled to be present at the GPs and nerves. In the PV areas, the ionic currents were adjusted as described by Cha et al. [10], in order to model the different ionic currents between the PV and LA.

### Three-dimensional simulation

The following 2 simulations were performed. In the first simulation, the ionic current conditions used in the 2D simulation were applied in the three-dimensional (3D) model to test whether the same behavior of AP observed in the 2D simulation could be observed in the 3D model. Pacing was applied at a location in the left superior PV with a PCL of 100 ms, from 0 to 7 s, to mimic the ectopic beats often observed in AF patients. Pacing was also applied at the location of Bachmann’s bundle, with a PCL of 1000 ms, from 0 to 20 s, to simulate normal beats. From 0 to 7 s, the 2 pacings were applied simultaneously to simulate PV triggers during a sinus rhythm. The action potentials at multiple points in the areas of the PV and LA were examined for any irregular depolarization. In the second simulation, fibrillatory wave patterns were examined for any triggered activity. To induce self-sustained AF, the ionic current conditions were adjusted as follows: I_K1_ was increased by 50%, whereas Ito, I_CaL_, and I_Kur_ were reduced by 80%, 40%, and 50%, respectively, from the conditions used by Courtemanche et al. [9]. These adjustments were made to sustain the AF for longer than 30 s based on previous studies of AF simulation [18, 19]. The AF was induced by rapid pacing at the location of Bachmann’s bundle. The action potentials at multiple points in the areas of the PV and LA were examined for any triggered activity. The map of the CFAE-cycle length (CFAE-CL) was obtained as described by Yun et al. [20]. The CFAE-CL was compared between the models with and without the ACh effect for each of the atrial models of 10 patients. The patient characteristics are shown in Table 1. Phase singularities (PSs) were also examined in the 3D AF model. PSs were identified every 1 ms for 6 s by using the method described by Iyer and Gray. [21] The number and duration of PSs were compared between the models with and without the ACh effect. To examine the effect of parasympathetic stimulation on the outcome of AF ablations, 3 patterns of virtual ablations were performed on sustained AF models of 10 patients with or without ACh stimulation: GP area ablation alone, circumferential PV isolation (CPVI) including the GP, and CPVI excluding the GP. The ablations were applied 6 s after the end of pacing. The wave patterns after ablation were observed for 30 s. No flux condition was applied to the ablated area. The simulations were performed using a customized software (CUVIA, ver. 1.0, Laonmed, Inc., Seoul, Korea).

**Table 1:** Patient characteristics.

Age, years	57.9 ± 13.6
Male (%)	90
Persistent AF (%)	70
CHA_2_DS_2_-VASc score	1.5 ± 1.9
Heart failure (%)	0
Hypertension (%)	30
Age >75 years (%)	10
Age 65–74 years (%)	10
Diabetes (%)	10
Previous stroke (%)	0
Previous TIA (%)	10
Vascular disease (%)	20
Echocardiographic findings	
LA dimension (mm)	44.8 ± 5.5
EF (%)	60.3 ± 12.2
E/Em	9.3 ± 4.2

AF, atrial fibrillation; TIA, transient ischemic attack; LA, left atrium; EF, ejection fraction; E/Em, the ratio of early diastolic mitral inflow velocity (E) to early diastolic mitral annular velocity (Em).

### Statistical analysis

Continuous variables were presented as the mean ± standard deviation and categorical variables were presented as a percentage of the group total. Data for GPs with or without GP stimulations were compared using the paired *t*-test for continuous variables, and the results were verified using the Wilcoxon signed-rank test. Categorical variables were compared using the Chi-square test as well as Fisher’s exact test, when necessary. A two-sided p-value of less than 0.05 was regarded as statistically significant. Statistical analysis was performed using SPSS (version 20.0, Statistical Package for Social Sciences, Chicago, IL, USA) software for Windows.

## Results

### Acetylcholine stimulates late phase 3 EAD from PV cells

**Fig 2:**
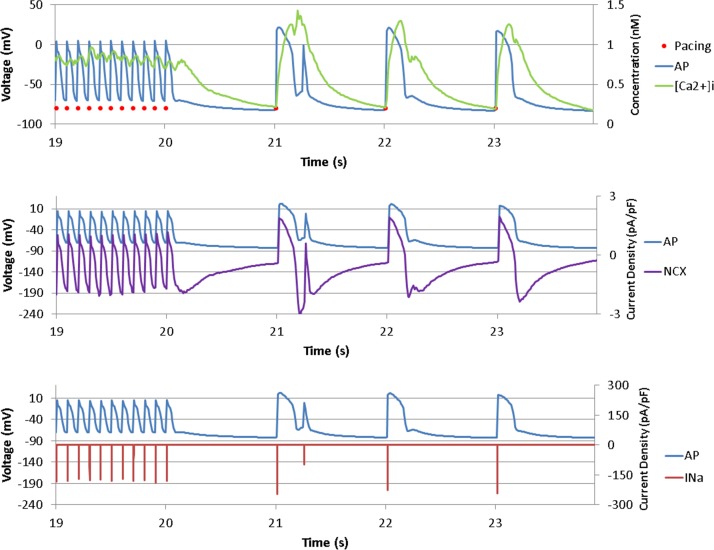
Late phase 3 early afterdepolarization (EAD)-like depolarization. Action potentials (APs), intracellular calcium concentration, sodium-calcium exchanger (NCX) current, and Na^+^ current before and after the pacing cycle length (PCL) change from 100 to 1000 ms are shown. A late phase 3 EAD-like depolarization is observed at the first beat after the PCL change. Increases in the intracellular calcium, NCX current, and Na^+^ current accompanied the late phase 3 EAD-like depolarization. The red dots represent the pacing time points.

Fig 2 shows the action potential, intracellular calcium concentration, and I_Na_ and NCX currents for ionic current conditions of 2D simulation described in the Methods section, where both sympathetic and parasympathetic stimulations were applied to the PV cells. When the PCL was changed from 100 to 1000 ms, the first beat after the PCL change exhibited a late phase 3 EAD-like depolarization. The intracellular calcium concentration, NCX current, and I_Na_ increased along with the depolarization. At the first beat after the PCL change, NCX removes Ca^2+^ while importing 3 Na^+^ at phase 3 repolarization. However, the [Ca^2+^]_i_ was still high, the membrane potential reached above the threshold for depolarization, and late phase 3 EAD-like depolarization occurred. The depolarization caused a spike in the NCX and removal of Na^+^. The depolarization was observed only at the first beat after the PCL change. The baseline potential was elevated to -71.0 mV at the end of pacing, with a PCL of 100 ms. The ranges of adjustments in ionic currents that resulted in abnormal depolarization are as follows: NCX: 2-3-fold, I_Na_: 10–27 mV shift in the inactivation curve, I_CaL_: 7-14-fold. Note that all these currents were adjusted under the condition of parasympathetic stimulation (0.05 μM of ACh). The ranges of the first and the second PCLs for which EAD was observed were 70-100ms and 830-1070ms, respectively.

### Autonomic nerve stimulates PV tachycardia and drivers during AF

**Fig 3:**
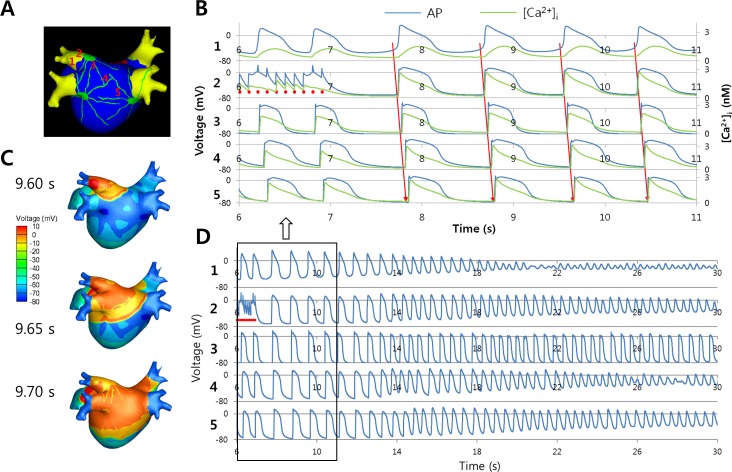
Spontaneous action potential in the pulmonary vein (PV). (A) Ganglionated plexus (GP) and nerve model based on the octopus hypothesis.[6] (B) Action potentials (APs) at 5 points on the PV and left atrium (LA) are shown after rapid pacing in the left superior PV (location 2). Sequential wave propagation from point 1 to 5 is observed. Red dots represent the pacing time points. (C) Three-dimensional AP maps showing wave propagation. (D) Approximately 7 s after rapid pacing, the wave propagation becomes irregular and degenerates to atrial fibrillation (AF).

The octopus model of the GP and nerves applied to the 3D model is shown in Fig 1. An ACh stimulation of 0.05 μM was applied to the GPs and parasympathetic nerves. The model was paced at the left superior PV (location 2 in Fig 3A) with a PCL of 100 ms for 7 s. Immediately after rapid PV pacing, spontaneous action potentials were generated from the PV, adjacent to the pacing site (location 1 in Fig 3A–3D), which finally degenerated to AF (Fig 3D). The spontaneous AP showed a gradual elevation of phase 4 depolarization and baseline membrane potential, with reduced dV/dt of phase 0 depolarization, thus mimicking sinus node activation (Fig 3B). The baseline [Ca^2+^]_i_ was slightly higher and the changes in [Ca^2+^]_i_ were also different at the location of spontaneous AP, compared with the area of passive conduction. This automatic PV tachycardia propagated and initiated reentry in the LA roof area at approximately 7 s after the end of the pacing, which resulted in wave-break and AF. We tested a range of the fast PCL and increases in the I_CaL_ for spontaneous APs, and observed spontaneous APs with a fast PCL of 10–380 ms and an increase in the I_CaL_ by at least 7-fold.

**Fig 4:**
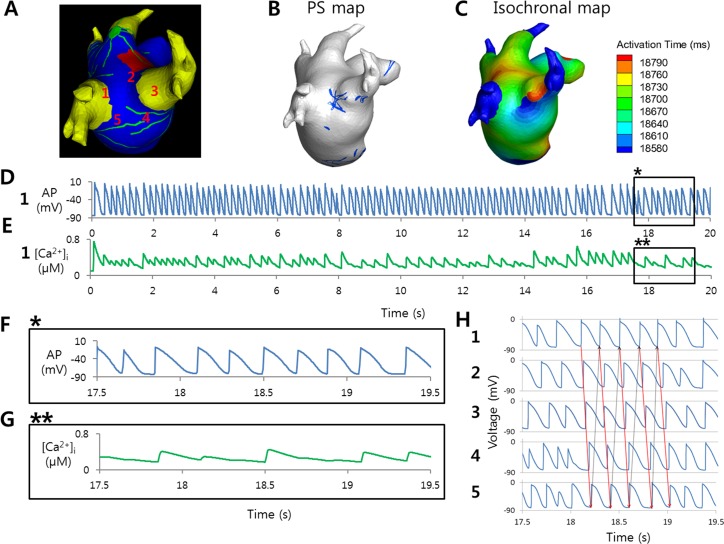
A local reentry in the maintenance of atrial fibrillation. (A) The location of reentry. The numbers indicate the locations of the action potentials (APs) shown in (H). (B) Phase singularity (PS) map showing the trajectory of the center of the reentry near the PV-LA junction. (C) Isochronal map showing the reentry near the PV-LA junction. (D) AP at a location (1 in (A)) in the reentry. (E) [Ca^2+^]_i_ at the location (1 in (A)) where AP in (D) was recorded. (F) Magnification of the box (marked *) in (D). (G) Magnification of the box (marked **) in (E). [Ca^2+^]_i_ did not always increase at each depolarization in (F). (H) APs at 5 locations (marked in (A)) around the reentry. APs exhibit the rotational propagation near the PV-LA junction.

Fig 4 shows the maintenance state of AF, which adopted the ionic current conditions of sustained AF and ANS stimulation. Intermittent local reentries were observed at the LA-PV junction during the maintenance of AF (S1 Movie). The rotor-like reentries without significant [Ca^2+^]_i_ changes suggest that the mechanism of local reentry may represent a potential driver of AF.

### GP stimulations change the AF wave dynamics

**Fig 5:**
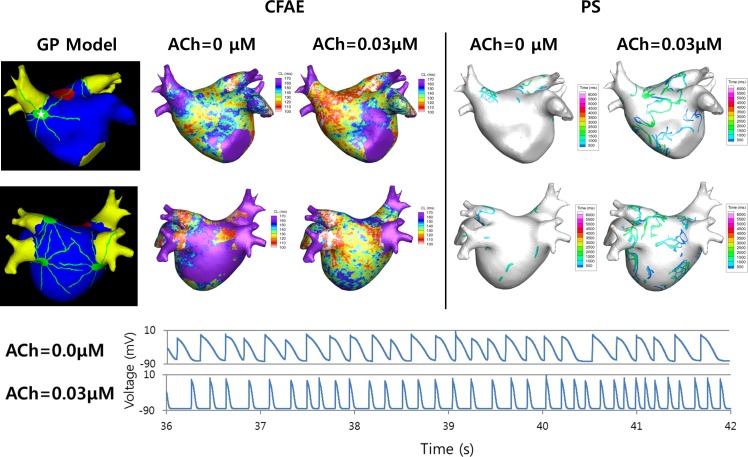
Wave-dynamics parameters following parasympathetic stimulation. Complex fractionated atrial electrogram (CFAE) and phase singularity (PS) maps for ACh concentrations of 0 and 0.03 μM are shown. The CFAE cycle length (CFAE-CL) is shorter overall, and more PSs are observed for an ACh concentration of 0.03 μM than 0 μM. The action potential (AP) at a point in the right anterior ganglionated plexus is shown for both ACh concentrations. The APD is shorter for ACh concentrations of 0.03 than 0 μM.

We conducted virtual CFAE-CL mapping and virtual PS mapping in 3D realistic AF models integrated by the LA geometries of 10 patients at the ionic current conditions of sustained AF. The patient characteristics are listed in Table 1. Fig 5 shows a representative example of virtual CFAE-CL and PS maps with or without GP stimulation (0.03 μM ACh). GP stimulation significantly reduced the CFAE-CL (p<0.01), and this change was more significant at the GP area than in the rest of the LA (p<0.01, Table 2). The degree of co-localization of the GP area with the CFAE area (CFAE-CL≤120ms) was more significant after GP stimulation (9.5% vs. 59.0%, Table 2). The number of PSs during the 6-s period of AF was greater (4552 vs. 9944), and the life span of PS was significantly shorter (66±100ms vs. 60±88ms, p<0.01) after GP stimulation in the specific example shown in Fig 5.

**Table 2:** CFAE-CL at 4 different GPs with or without GP stimulations (10 patients).

	Control	GP stimulation	Δ(GP stimulation–Control)	p-value
CFAE-CL at GP areas (ms)	138±5	117±4	-20±7	< 0.01
RAGP (ms)	140±11	118±7	-22±15	< 0.01
LSGP (ms)	135±10	114±9	-20±12	< 0.01
LIGP (ms)	139±8	117±11	-23±13	< 0.01
RIGP (ms)	140±11	119±9	-21±14	< 0.01
CFAE-CL at non-GP areas (ms)	135±2	127±4	-9±4	< 0.01
Proportions of CFAE areas† at GP areas	9.5±4.3*	59.0±11.8**	49.5±14.1	< 0.01
RAGP (%)	8.9±13.6	58.9±21.6	49.9±25.1	< 0.01
LSGP (%)	14.9±16.4	66.8±24.7	51.9±32.2	< 0.01
LIGP (%)	4.6±3.4	58.4±28.4	53.8±27.8	< 0.01
RIGP (%)	7.8±8.7	61.0±27.0	53.1±27.2	< 0.01
Proportions of CFAE areas at non-GP areas	10.0±2.6*	24.7±5.4**	14.8±5.9	< 0.01

†CFAE area is defined as the area where the CFAE is < 120 ms.

*Under control conditions, the proportions of CFAE areas in GP areas are statistically not different from those in the non-GP areas (p = 0.62).

**Under GP stimulation conditions, the proportions of CFAE areas in GP areas are significantly higher than those in the non-GP areas (p < 0.01).

CFAE, complex fractionated atrial electrogram; CL, cycle length; GP, ganglionated plexus; RAGP, right anterior ganglionated plexus; LSGP, left superior ganglionated plexus; LIGP, left inferior ganglionated plexus; RIGP, right inferior ganglionated plexus.

**Fig 6:**
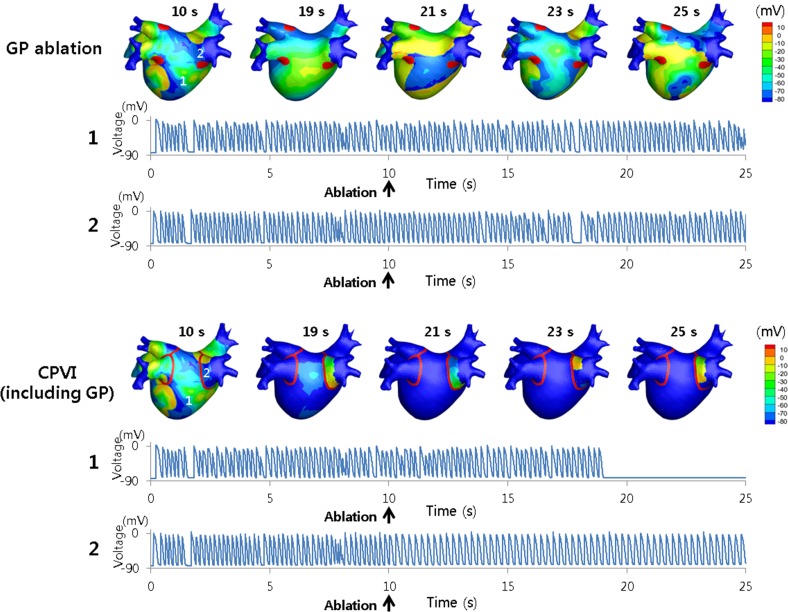
Two patterns of virtual ablations. Ganglionated plexus (GP)-only ablation did not terminate atrial fibrillation (AF), whereas circumferential pulmonary vein isolation (CPVI) including the GP areas terminated AF at approximately 9 s after the virtual ablation was performed. In the case of CPVI including the GP areas, activity was still observed within the ablation line around the right inferior pulmonary vein (PV). Action potentials (APs) in the left atrium (LA) and PV are shown for both patterns of virtual ablations.

Table 3 shows the results of different ablations with or without ACh stimulation. Among the ablation strategies, CPVI including the GP was the most effective whereas GP-only ablation was the least effective. CPVI excluding the GP was less effective in the presence of ACh stimulation, as compared to no ACh stimulation. Fig 6 shows representative examples of action potential distributions with time for GP-only ablation and CPVI including the GP.

**Table 3:** The results of 4 ablations in 10 patient models during 30 s after ablation.

	GP-only ablation with ACh stim.	CPVI+GP ablation with ACh stim.	CPVI-only with ACh stim.	CPVI only without ACh stim.
**Patient 1**	AF	**AT (18.5s)**	AF	**AT (13.6s)**
**Patient 2**	AF	**AT (11.7s)**	AF	**AT (6.9s)**
**Patient 3**	AF	**Term (6.4s)**	AF	**AT (11.4s)**
**Patient 4**	AF	**Term (24.5s)**	AF	**AT (19.4s)**
**Patient 5**	AF	**Term (1.3s)**	**AT (7.4s)**	**AT (9.6s)**
**Patient 6**	AF	**AT (6.4s)**	**AT (24.8s)**	**AT (12.1s)**
**Patient 7**	**AT (20.3s)**	**Term (7.5s)**	**AT (10.8s)**	**AT (10.7s)**
**Patient 8**	AF	**AT (5.3s)**	AF	AF
**Patient 9**	AF	**Term (16.6s)**	AF	AF
**Patient 10**	AF	**Term (9.1s)**	AF	**AT (11.0s)**
**Summary**	**Term: 0**	**Term: 6**†	**Term: 0**	**Term: 0**
	**AT: 1**	**AT: 4**	**AT: 3**	**AT: 8**††
	AF: 9*	**AF: 0**	AF: 7**	**AF: 2**

AF = AF maintained. AT = Converted to AT. Term = AF terminated. The number in parenthesis is the time from ablation to AF termination or conversion to AT.

†p = 0.01 compared with GP-only ablation with ACh stimulation, CPVI-only with ACh stimulation, or CPVI-only without ACh stimulation.

††p<0.01 compared with GP-only ablation with ACh stimulation.

*p<0.01 compared with CPVI+GP ablation with ACh stimulation or CPVI-only without ACh stimulation.

**p<0.01 compared with CPVI+GP ablation with ACh stimulation.

## Discussion

In the present study, we incorporated the ANS model into an *in silico* 3D realistic model of AF. We observed that cardiac ANS stimulations provoked triggered activity, PV automaticity, and local reentries at the LA-PV junction in this simulation model. The parameters reflecting cardiac wave dynamics, such as CFAE and PS, were significantly affected by virtual GP stimulation, and showed co-localization of the GP and CFAE areas.

### Role of the autonomic nerve in AF triggers

One of the several mechanisms by which the ANS triggers AF is late phase 3 EAD, which occurs when the APD is shortened and the Ca^2+^ transient is increased [4]. Vandersickel et al. [22] showed that abnormal wave patterns emerged from EAD in a mathematical model for human ventricular cardiac tissue. The sympathetic nervous system increases the Ca^2+^ transient, and the parasympathetic nervous system activates I_K[ACh]_, which shortens the APD. The late phase 3 EAD-like depolarization observed in the 2D model of the current study was generated under conditions of increased I_CaL_. An increase in the Ca^2+^ release current from the sarcoplasmic reticulum release compartment (I_rel_) or Ca^2+^ leak current from the sarcoplasmic reticulum uptake compartment (I_leak_) in the Courtemanche et al. [9] model, without any change in I_CaL_, did not lead to any irregular depolarization. The NCX seems to play an important role in the occurrence of late phase 3 EAD. Burashnikov and Antzelevitch [5] explained the mechanism in terms of a strong inward NCX and calcium current, as the intracellular calcium peaked in the late phase of repolarization when APD was shortened. In the current study, the increases in I_CaL_ and NCX, and the presence of ACh were not sufficient to induce any irregular depolarization. By using an *in silico* model, Morotti et al. [12] showed that EADs can be prevented by ranolazine, which reduces atrial peak I_Na_. The reverse effect of ranolazine was applied to the current model by shifting the inactivation curve of I_Na_ by +10 mV, thereby widening the voltage range of Na^+^ influx. On the other hand, the spontaneous action potentials in the PV region close to the rapid pacing site in the 3D model seem to have resulted from the elevated baseline potential due to the accumulated intracellular calcium. PV regions are known to often harbor the sites of ectopic beats. We also ran the simulation without ACh, and spontaneous APs were still observed. However, when the I_CaL_ was not increased, spontaneous APs were not observed. Chou et al. [23] experimentally observed that an infusion of ryanodine and isoproterenol resulted in focal discharges from PVs. Experimental studies showed that perfusion of isoproterenol increased I_CaL_ [13, 14].

### Roles of the autonomic nerve in AF maintenance

AF-triggering activities such as EADs and delayed afterdepolarizations, which can be induced by the ANS, occur during the progression of AF and can contribute to the maintenance of AF. The parasympathetic nervous system, in particular, has clusters of neurons (GPs) on the atrium, and the ACh concentration exhibits a spatially heterogeneous distribution [4]. As a result, the APD distribution becomes heterogeneous, which makes the cardiac wave unstable. Sarmast et al. [24] showed that the higher I_K[ACh]_ density in LA than in right atrium (RA) myocytes resulted in the generation of a larger number of rotors, as well as an increase in the frequency and number of rotations of the rotors in the LA than in the RA in sheep hearts. The larger number of PSs generated and their shorter duration observed in the present study in the case of parasympathetic stimulation indicate the presence of more unstable cardiac waves. A large number of PSs indicates large number of reentries generated. Reentries have been linked to persistent AF in several studies [25, 26]. Fig 4 shows an example that indicates that a triggering activity generated in the progression of AF corresponds to a reentry near GP. Another cardiac wave dynamics parameter of interest is CFAE, which represents the electrogram cycle length. The CFAE has been a frequent target in the procedure of catheter ablation for AF. CFAE has been studied extensively since Nademani et al. [27] proposed CFAE as a potential ablation target. Although controversies exist regarding the efficacy of CFAE ablation [28], it remains as one of the ablation strategies used by many physicians. In the present study, CFAE-CL was significantly shorter in the case of ACh stimulation. A shorter CFAE-CL indicates more fractionations in the electrograms, which in turn indicates more chaotic wave propagation patterns. Moreover, ACh stimulation decreased CFAE-CL more in the GP areas as compared to the non-GP areas (Table 2), which implies that the GP areas could be potential targets for ablation. The decreased CFAE-CL in the presence of GP stimulation can be attributed to decreased APD due to ACh and the heterogeneous distribution of APD, as well as repeated activation sources from the PV or the PV-LA junction. The higher percentage of CFAE area in the GP in the case of parasympathetic stimulation confirms the effect of APD and its distribution. Parasympathetic stimulation leads to heterogeneous CFAE distribution (higher percentage in the GP), which translates to unstable cardiac waves.

### Can GP ablation reduce AF?

Radiofrequency catheter ablation has become a common procedure for AF, and appropriate targets for ablation are being constantly researched. The observation in this study that CPVI including the GP areas yielded superior outcomes as compared to GP-only ablation is consistent with the clinical findings. GP ablation combined with PV isolation has been found to be effective, but GP-only ablation was not very effective [29]. The outcome of GP + PV ablation was also superior to PV isolation alone [29]. This suggests that GP ablation eliminates the sources of AF other than the ectopic beats from the PV. GP ablation would eliminate the heterogeneity of APD distribution caused by ACh. In addition, GP ablation may affect the electrophysiology down-stream of the nerve endings spread along the entire atrium, and may remove a large portion of the CFAE area. However, GP-only ablation would not remove the ectopic beats from the PV, as mentioned above, as well as non-PV sources such as rotors. Moreover, it is difficult to ablate GPs with endocardial ablation alone, as GPs exist on the epicardial aspect of the LA-PV junction. Because the mechanisms of AF initiation and maintenance are multifactorial, GP ablations would need to be accompanied by ablations targeting areas other than GPs.

### Limitations

This study has some limitations. As the detailed anatomical distribution of the autonomic nerves in the atrium is unknown, the nerve structure used in this study was based on a hypothesis. GP and cardiac nerves were modeled by applying the same ionic currents, but with different morphologies. The 3D atrial model used in this study is a structurally homogeneous LA model that excluded the histological features. Thickness variation and structural characteristics such as fiber orientation and fibrosis would affect wave propagation. The addition of the RA can also change the wave dynamics, although AF drivers are known to exist mostly in the LA. The ionic current properties are spatially uniform in this model. The spatial heterogeneity in the ionic currents is known to play a major role in rotor drifting and peripheral wave breakups.

## Conclusion

Simultaneous GP and I_Ca-L_ stimulation induced triggered activity from the LA-PV junction and PV tachycardia, thus changing the wave dynamics of the AF maintenance mechanism in a 3D *in silico* GP model of the LA.

## Supporting information

S1 Movie Local reentry at the PV-LA junction. (WMV)
